# Evaluation of neoadjuvant chemotherapy followed by radical hysterectomy in cervical cancer: a single-center study

**DOI:** 10.1007/s10147-026-02998-0

**Published:** 2026-03-04

**Authors:** Akitoshi Yamamura, Masayo Ukita, Hiromi Takemura, Tetsuya Ishibashi, Hinako Nakanishi, Mariko Kita, Takaya Sakamoto, Airi Toda, Teruki Yoshida, Sayuri Takahashi, Shota Kanbayashi, Hirohiko Tani, Kenzo Kosaka

**Affiliations:** Department of Obstetrics and Gynecology, Shizuoka General Hospital, 4-27-1 Kita Ando, Aoi-Ku, Shizuoka City, Shizuoka 420-8527 Japan

**Keywords:** Cervical cancer, Neoadjuvant chemotherapy, Radical hysterectomy, Propensity score matching, Inverse probability weighting

## Abstract

**Background:**

The efficacy of neoadjuvant chemotherapy (NACT) before radical hysterectomy (RH) in cervical cancer remains unclear. We evaluated stage-specific outcomes of NACT-RH at a high-volume center.

**Methods:**

This retrospective cohort study included patients with non-metastatic cT1b1–2b cervical cancer who underwent RH between 2013 and 2022. For cT1b cases, prognostic outcomes were compared between patients with cT1b3 (any N) who underwent NACT-RH (High-risk, NACT(+)) and those with cT1b1–1b2 N0 who underwent primary RH (Low-risk, NACT(−)), using propensity score matching. For cT2 cases, NACT-RH versus primary RH was compared using inverse probability weighting to adjust for baseline differences.

**Results:**

Among 191 patients who underwent RH (cT1: *n* = 99; cT2: *n* = 92), the matched cT1 cohort comprised 15 High-risk, NACT(+) and 30 Low-risk, NACT(−) patients. Five-year progression-free survival (PFS) was 75.0% (95% confidence interval [CI], 53.4–100.0%) versus 89.9% (95% CI, 79.6–100.0%) (*p* = 0.427). Overall survival (OS) was 100% in both groups with a median follow-up of > 70 months. In cT2, NACT-RH (*n* = 73) versus primary RH (*n* = 19) showed hazard ratios of 2.88 for PFS (95% CI, 0.69–11.97) and 5.64 for OS (95% CI, 0.70–45.35) after baseline adjustment. Among cT2 patients who underwent NACT-RH, PFS and OS were worse in those without an objective response to NACT (*n* = 18, all cT2b) than in responders.

**Conclusion:**

NACT-RH was associated with encouraging long-term outcomes in cT1b3, whereas outcomes in cT2 cases remain uncertain, supporting careful selection and response-adapted strategies.

**Supplementary Information:**

The online version contains supplementary material available at 10.1007/s10147-026-02998-0.

## Introduction

Cervical cancer remains a common gynecologic malignancy worldwide, with approximately 600,000 new cases reported annually [1]. The principal definitive treatment options for locally advanced cervical cancer include radical hysterectomy (RH) and concurrent chemoradiotherapy (CCRT). The treatment strategy is determined by integrating multiple factors, including disease stage, tumor size, patient age, comorbidities, and institutional resources.

Regarding surgical management, Okabayashi RH has been performed in Japan for decades. In the Okabayashi RH, the vesico-uterine ligament is divided into anterior and posterior leaves, and the posterior leaf is resected adequately; the vagina and paracolpium are also widely excised, which is expected to provide favorable local control [2, 3]. Accordingly, across multiple institutions in Japan, RH has long been positioned as an important treatment option for patients with T1b3–2b disease (UICC 9th edition) [4].

Among patients with locally advanced cervical cancer for whom RH is considered, achieving adequate surgical margins may occasionally be challenging, particularly in cases with bulky tumors or suspected parametrial involvement. To facilitate complete resection by reducing tumor volume and local extent, neoadjuvant chemotherapy (NACT) has been used as a preoperative strategy in such settings [5]. NACT has been reported to decrease the incidence of parametrial involvement and lymph node metastasis, and is therefore regarded as a theoretically attractive treatment approach [6].

However, the impact of NACT on prognosis remains inconclusive. While some studies have suggested that adding NACT may reduce recurrence and potentially improve overall survival (OS) [7], a Japanese phase III randomized controlled trial (RCT) reported no additional OS benefit of NACT followed by RH compared with primary RH [8]. These discrepant findings may reflect heterogeneity in multiple factors, including the distribution of disease stage, NACT regimens, surgical technique, the use and type of postoperative adjuvant therapy, and inter-institutional differences in the quality of RH.

From the perspective of comparing surgery with CCRT, for patients with locally advanced cervical cancer corresponding to T1b3–2b disease (UICC 9th edition), RH following NACT (NACT-RH) has been shown to yield inferior disease-free survival (DFS) compared with CCRT [9]. In light of this evidence, the National Comprehensive Cancer Network (NCCN) and Japanese clinical practice guidelines position NACT-RH as a treatment strategy that is generally not recommended as a standard approach [10, 11].

However, outcomes following RH have been reported to vary across institutions [12], and high-volume centers are considered to achieve better local control and DFS [13]. In this context, NACT-RH performed at high-volume centers may still represent a clinically reasonable treatment option, despite its low level of recommendation in current guidelines. Nevertheless, data from Japanese high-volume centers regarding the stage-specific effectiveness of NACT-RH remain limited.

Accordingly, this single-center study conducted at a high-volume institution aimed to evaluate the clinical outcomes of NACT-RH according to clinical stage. Specifically, we sought to: (1) contextualize long-term outcomes after NACT-RH in cT1b3 (any N) disease by comparing them with a lower-risk reference cohort (cT1b1–1b2 cN0) treated with primary RH, acknowledging that this comparison is exploratory and not intended to imply treatment equivalence; and (2) compare progression-free survival (PFS) and OS among patients with cT2 disease according to whether NACT was administered, thereby assessing the validity of a NACT-RH strategy in this setting. Through these analyses, we aimed to clarify the indications and limitations of NACT-RH in a high-volume center and to provide evidence that may help optimize treatment strategies in clinical practice.

## Patients and methods

### Study design and population

This retrospective cohort study was conducted at Shizuoka General Hospital. Patients with cervical cancer who initiated first-line treatment between April 1, 2013, and December 31, 2022, were identified from the electronic medical records. The data cutoff date was February 28, 2025. Eligible patients had non-metastatic cervical cancer classified as cT1b1–2b (UICC 9th edition), regardless of histologic subtype. Patients whose initial treatment was radiotherapy (RT) alone or CCRT were excluded.

This study was approved by the institutional ethics committee (SGHIRB#2025010). In accordance with institutional policy, study information was disclosed on the hospital website and an opt-out option was provided. The study was conducted in compliance with the Declaration of Helsinki and relevant Japanese laws and ethical guidelines.

### Diagnostic work-up and treatment protocol

The diagnostic work-up was based on pelvic examination, transvaginal ultrasonography, histopathologic confirmation, pelvic magnetic resonance imaging (MRI), and computed tomography (CT) of the chest, abdomen, and pelvis, with diagnostic conization performed when indicated. The maximum tumor diameter was measured on MRI. In principle, patients with non-metastatic cT1b1–2b disease underwent Okabayashi RH. Pelvic lymphadenectomy was performed in all patients, and para-aortic lymphadenectomy was additionally performed when para-aortic metastasis was suspected on CT. A nerve-sparing approach was routinely attempted; however, nerve sparing was omitted on the affected side when parametrial involvement was suggested by pelvic examination, imaging findings, or intraoperative assessment. When parametrial involvement was considered to have resolved after NACT, ipsilateral nerve-sparing was permitted.

Indications for NACT were primarily based on a tumor diameter of > 40 mm, and eligibility was determined after discussion at an institutional conference. Patients with squamous cell carcinoma received irinotecan (60 mg/m^2^) plus nedaplatin (80 mg/m^2^), whereas those with adenocarcinoma received carboplatin (target area under the concentration–time curve [AUC] 5) plus paclitaxel (175 mg/m^2^) or cisplatin (50 mg/m^2^) plus paclitaxel (67.5 mg/m^2^), as determined by the treating physician. NACT was administered every 3 weeks for two cycles, followed by RH. Postoperatively, the same regimen was planned for an additional four cycles. Regimen modification was permitted according to treatment response and toxicity.

For patients who did not receive NACT, postoperative adjuvant therapy was administered when any of the following high-risk features were present: positive surgical margins, lymph node metastasis, or parametrial involvement. In addition, the need for adjuvant therapy was discussed for each patient based on tumor size, depth of stromal invasion, and the presence of lymphovascular space invasion (LVSI). In Japan, chemotherapy is used as an alternative to RT/CCRT for postoperative adjuvant treatment in approximately 70% of institutions [14], and a phase III RCT is currently underway to evaluate this approach [15]. Given this practice pattern, adjuvant treatment in our cohort primarily consisted of chemotherapy.

After completion of the initial treatment, patients were followed using a combination of pelvic examination, cytology of the vaginal cuff, transvaginal ultrasonography, tumor markers, and CT imaging.

### Endpoints and definitions

The primary endpoints were PFS and OS in patients with cT1b3 (any N) disease who underwent NACT-RH, defined as the High-risk, NACT(+) group. In our institution, NACT-RH was the standard initial approach for cT1b3 disease; therefore, there were only three cT1b3 patients who underwent primary RH, making a meaningful within-stage comparison difficult (cT1b3 NACT(+) vs. cT1b3 NACT(−)). Accordingly, we selected as the control group patients with cT1b1–1b2 and cN0 disease who underwent primary RH (Low-risk, NACT(−) group), a cohort expected to have a more favorable baseline prognosis, to provide a clinically interpretable reference for outcomes after NACT-RH in cT1b3 disease. Importantly, because cT1b3 (any N) and cT1b1–1b2 cN0 represent structurally different biological risk profiles (tumor burden and nodal risk), this comparison is intended as a contextual benchmark rather than evidence of treatment equivalence, and any inference of comparable efficacy remains exploratory.

The secondary endpoints were PFS and OS in patients with cT2 disease, comparing NACT-RH versus primary RH. The exploratory endpoints were (1) post-recurrence treatment course in the High-risk, NACT(+) group and (2) PFS and OS in cT2 patients treated with NACT-RH according to NACT response.

For PFS, time zero was defined as the start date of NACT in patients treated with NACT-RH and the date of surgery in those treated with primary RH; recurrence was considered the event. For OS, death from any cause was considered the event. Local recurrence was defined as recurrence within the pelvis, and distant recurrence as metastasis outside the pelvis. NACT response was assessed on pre- and post-NACT MRI according to RECIST version 1.1; objective response was defined as complete response or partial response.

### Statistical analysis

Statistical analyses were performed using R (version 4.1.1; R Foundation for Statistical Computing, Vienna, Austria). Continuous variables were compared using Student’s t-test, and categorical variables were compared using Fisher’s exact test. The median follow-up time was estimated using the reverse Kaplan–Meier method.

For the analysis of NACT-RH in patients with cT1b3 disease, propensity scores were estimated using a logistic regression model including age and histology as covariates. Nearest-neighbor matching at a 1:2 ratio was performed with a caliper width of 0.2 to select matched controls from patients with cT1b1–1b2 and N0 disease. Kaplan–Meier curves were generated, and between-group differences were assessed using the log-rank test.

For comparisons among patients with cT2 disease, adjustment was performed by estimating the average treatment effect on the treated using inverse probability weighting (ATT-IPW) with unstabilized weights, incorporating age, histology, cT category, and cN status. Weights were winsorized at the 1st and 99th percentiles. Weighted Kaplan–Meier curves were generated, and Cox proportional hazards models included cT and cN as covariates because their standardized mean differences (SMDs) remained > 0.1 after weighting.

As a sensitivity analysis to evaluate the impact of potential immortal time bias in the NACT group, we redefined time zero as the date of surgery for both the NACT and non-NACT cohorts and repeated the survival analyses accordingly.

For comparisons of PFS and OS by NACT response among cT2 patients treated with NACT-RH, unadjusted Kaplan–Meier analyses were performed and compared using the log-rank test.

One cT2 patient lacked post-NACT MRI tumor-size data and was therefore excluded from the cT2 subgroup analysis of response to NACT. No other variables had missing values; accordingly, multiple imputation was not performed. All statistical tests were two-sided, and a *p*-value < 0.05 was considered statistically significant.

## Results

### Patient population

**Fig. 1:**
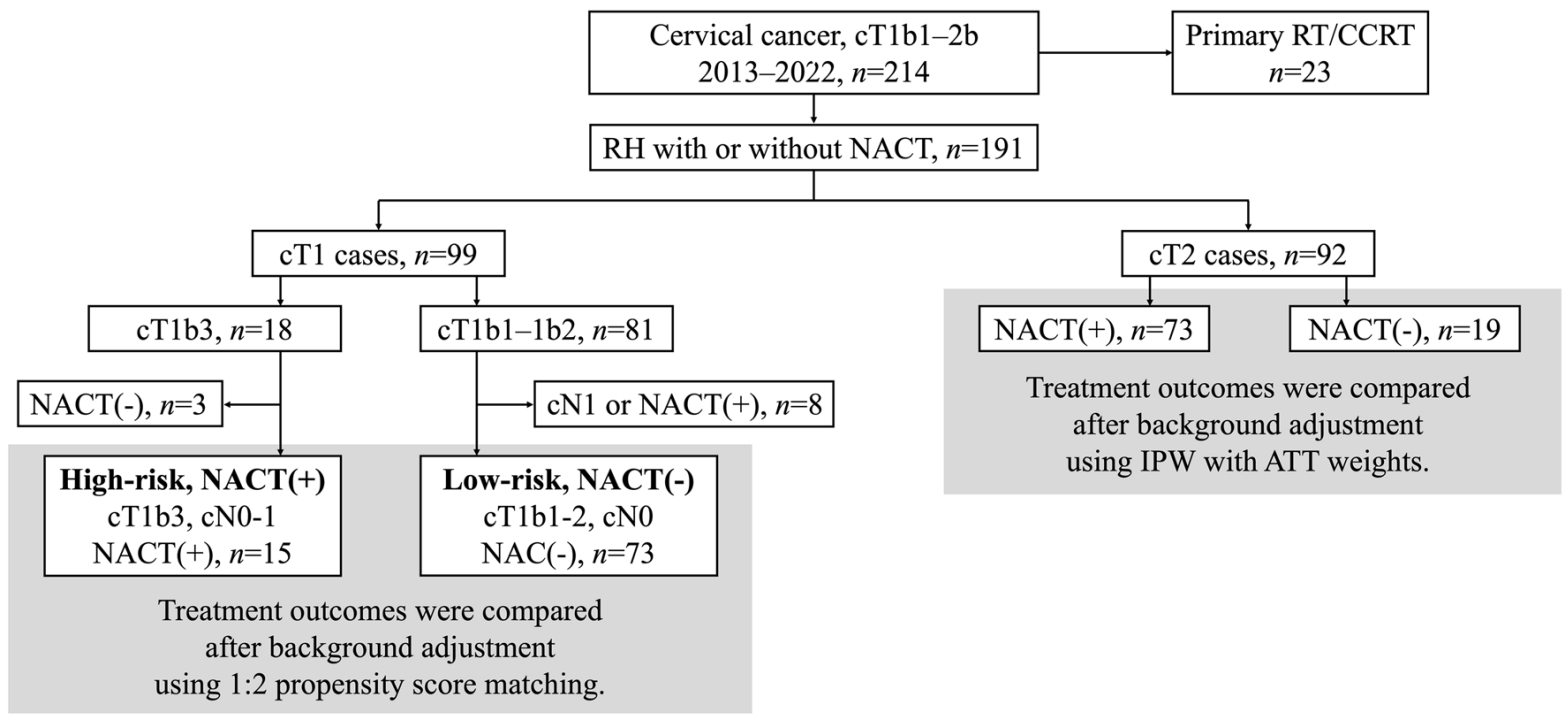
Study Flow Diagram. Patients were analyzed according to the flowchart. Of 214 patients with cT1b1–2b cervical cancer, 23 initially treated with RT/CCRT were excluded. The remaining 191 patients who underwent RH were analyzed: 99 cT1 and 92 cT2. In the cT1 cohort, 18 had cT1b3 (15 NACT-RH) and 81 had cT1b1–1b2 (73 cN0 primary RH). In the cT2 cohort, 73 underwent NACT-RH and 19 primary RH. *RH* radical hysterectomy, *RT* radiotherapy, *CCRT* concurrent chemoradiotherapy, *NACT* neoadjuvant chemotherapy

Among 214 patients with cT1b1–2b cervical cancer during the study period, 23 patients whose initial treatment was RT or CCRT were excluded. The remaining 191 patients who underwent RH were included in the analysis (Fig. 1). Of these, 99 had cT1 disease and 92 had cT2 disease.

Within the cT1 cohort, 18 patients had cT1b3 disease, 15 of whom underwent NACT-RH, and 81 patients had cT1b1–1b2 disease, including 73 who were N0 and underwent primary RH. Within the cT2 cohort, 73 patients underwent NACT-RH and 19 underwent primary RH.

## 1) cT1b analysis (High-risk, NACT(+) vs. Low-risk, NACT(−); propensity score-matched cohort)

### Baseline characteristics

We first analyzed patients with cT1b disease. After propensity score matching (1:2 nearest-neighbor matching; covariates: age and histology), the matched cohort comprised 15 patients in the High-risk, NACT(+) group and 30 patients in the Low-risk, NACT(−) group (Table 1). The between-group differences in age and histology observed before matching were attenuated after matching. In the matched Low-risk, NACT(−) group, pT2b accounted for 6.7% (2/30) of patients. LVSI was observed in 6.7% (1/15) of the High-risk, NACT(+) group and 26.7% (8/30) of the Low-risk, NACT(−) group. In the Low-risk, NACT(−) group, two patients had positive surgical margins. Both patients had pT2b disease: one had a positive vaginal margin, and the other had a positive parametrial margin.

**Table 1:** Baseline characteristics and surgical details of patients with cT1 disease before and after matching

	Before matching			After matching		
	High-risk, NACT (+) *n* = 15	Low-risk, NACT (−) *n* = 73	SMD	High-risk, NACT (+) *n* = 15	Low-risk, NACT (−) *n* = 30	SMD
Age^a^, year	49.3 ± 10.2	52.1 ± 14.8	0.224	49.3 ± 10.2	48.0 ± 10.7	0.122
Histology^a^						
SCC	10 (66.7)	43 (58.9)	0.161	10 (66.7)	18 (60.0)	0.139
Other	5 (33.3)	30 (41.1)		5 (33.3)	12 (40.0)	
cT						
1b1	0 (0.0)	31 (42.5)	1.646	0 (0.0)	17 (56.7)	1.237
1b2	0 (0.0)	42 (57.5)		0 (0.0)	13 (43.3)	
1b3	15 (100.0)	0 (0.0)		15 (100.0)	0 (0.0)	
cN						
0	9 (60.0)	73 (100.0)	1.155	9 (60.0)	30 (100.0)	1.155
1	6 (40.0)	0 (0.0)		6 (40.0)	0 (0.0)	
2	0 (0.0)	0 (0.0)		0 (0.0)	0 (0.0)	
PS						
0	15 (100.0)	72 (98.6)	0.167	15 (100.0)	30 (100.0)	< 0.001
1	0 (0.0)	1 (1.4)		0 (0.0)	0 (0.0)	
Tumor size on MRI, mm	56.4 ± 9.1	25.4 ± 9.3	3.365	56.4 ± 9.1	25.7 ± 10.0	3.218
Nerve sparing						
None	1 (6.7)	2 (2.7)	0.385	1 (6.7)	2 (6.7)	0.263
Unilateral	0 (0.0)	4 (5.5)		0 (0.0)	1 (3.3)	
Bilateral	14 (93.3)	67 (91.8)		14 (93.3)	27 (90.0)	
(y)pT						
0	1 (6.7)	0 (0.0)	0.974	1 (6.7)	0 (0.0)	1.165
1a1	0 (0.0)	1 (1.4)		0 (0.0)	0 (0.0)	
1a2	0 (0.0)	2 (2.7)		0 (0.0)	1 (3.3)	
1b1	3 (20.0)	30 (41.1)		3 (20.0)	16 (53.3)	
1b2	4 (26.7)	28 (38.4)		4 (26.7)	9 (30.0)	
1b3	4 (26.7)	6 (8.2)		4 (26.7)	2 (6.7)	
2a1	1 (6.7)	2 (2.7)		1 (6.7)	0 (0.0)	
2a2	1 (6.7)	0 (0.0)		1 (6.7)	0 (0.0)	
2b	1 (6.7)	4 (5.5)		1 (6.7)	2 (6.7)	
(y)pN						
0	12 (80.0)	68 (93.2)	0.393	12 (80.0)	30 (100.0)	0.707
1	3 (20.0)	5 (6.8)		3 (20.0)	0 (0.0)	
2	0 (0.0)	0 (0.0)		0 (0.0)	0 (0.0)	
LVSI	1 (6.7)	22 (30.1)	0.636	1 (6.7)	8 (26.7)	0.557
Positive surgical margins	0 (0.0)	2 (2.7)	0.237	0 (0.0)	2 (6.7)	0.378
Pathological tumor size, mm	30.3 ± 17.9	28.2 ± 13.2	0.132	30.3 ± 17.9	28.5 ± 13.0	0.115
Adjuvant						
None	0 (0.0)	41 (56.2)	1.646	0 (0.0)	19 (63.3)	1.859
Chemotherapy	15 (100.0)	31 (42.5)		15 (100.0)	11 (36.7)	
CCRT	0 (0.0)	1 (1.4)		0 (0.0)	0 (0.0)	
VD						
None	8 (53.3)	45 (61.6)	0.523	8 (53.3)	20 (66.7)	0.525
Medication only	6 (40.0)	20 (27.4)		6 (40.0)	9 (30.0)	
Temporary CIC	0 (0.0)	6 (8.2)		0 (0.0)	1 (3.3)	
Permanent CIC	1 (6.7)	2 (2.7)		1 (6.7)	0 (0.0)	
Follow-up period, month	70 [33–98]	65 [50–90]	0.029	70 [33–98]	74 [61–96]	0.239

Categorical valuables were presented as *n* (%). Follow-up periods were presented as median [IQR], and all other continuous valuables were presented as mean ± SD

*IPW* inverse probability weighting, *NACT* neoadjuvant chemotherapy, *SMD* standardized mean difference, *SCC* squamous cell carcinoma, *PS* performance status, *MRI* magnetic resonance imaging, *LVSI* lymphovascular space invasion, *CCRT* concurrent chemoradiotherapy, *VD* voiding dysfunction, *CIC* clean intermittent catheterization, *IQR* interquartile range, *SD* standard deviation

^a^Covariates used for propensity score matching

### PFS and OS

**Fig. 2:**
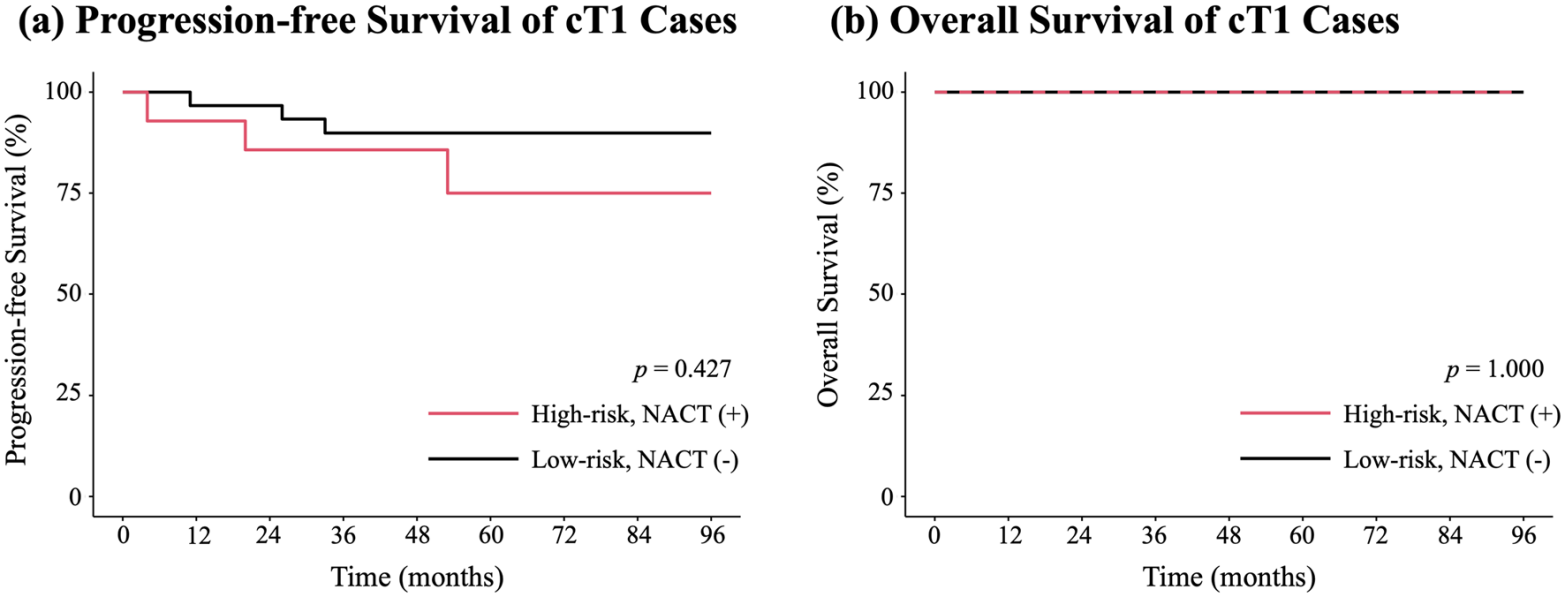
Kaplan–Meier Curves for cT1 Cases After Propensity Score Matching. **a** Progression-free Survival of cT1 Cases. After propensity-score matching, there was no significant difference between the High-risk, NACT(+) and Low-risk, NACT(−) groups. The 5-year progression-free survival was 75.0% in the High-risk, NACT(+) group and 89.9% in the Low-risk, NACT(−) group. **b **Overall Survival of cT1 Cases. Overall survival was 100% in both groups. *NACT* neoadjuvant chemotherapy

Kaplan–Meier curves were generated for the propensity score-matched cohort (Fig. 2). There was no significant difference in PFS between groups (*p* = 0.427). The 5-year PFS was 75.0% (95% confidence interval [CI], 53.4–100.0%; 3/15 events) in the High-risk, NACT(+) group and 89.9% (95% CI, 79.6–100.0%; 3/30 events) in the Low-risk, NACT(−) group. OS remained 100% in both groups over a median follow-up of more than 70 months (Fig. 2b). The sensitivity analysis in which time zero was set to the date of surgery for both groups is shown in Supplementary Fig. S1, and yielded results similar to those of the primary analysis.

### Post-recurrence course

The post-recurrence clinical course in the High-risk, NACT(+) group is summarized in Supplementary Fig. S2. Among the three patients who experienced recurrence, one had a local recurrence and two had distant recurrences. The local recurrence was controlled surgically. Of the two patients with distant recurrence, one underwent resection for a solitary lung metastasis, and the other received chemotherapy. As shown in Fig. 2b, no deaths occurred among these patients during follow-up.

## 2) cT2 analysis (NACT(+) vs. NACT(−); ATT-IPW cohort)

### Baseline characteristics

In the cT2 cohort, the NACT(+) group comprised 73 patients, whereas the control NACT(−) group included only 19 patients. To improve baseline comparability while retaining all available patients, ATT-IPW was applied, using propensity scores based on age, histology, cT category, and cN status (Table 2). Before weighting, there was substantial imbalance in cT stage, with T2b in 87.7% (64/73) of the NACT(+) group versus 42.1% (8/19) of the NACT(−) group, and cN positivity also tended to be more frequent in the NACT(+) group. After weighting, balance for age and histology was generally improved; however, residual imbalance remained for cT (SMD = 0.137) and cN (SMD = 0.373).

**Table 2:** Baseline Characteristics and Surgical Details of Patients with cT2 Disease Before and After IPW

	Unweighted			Weighted		
	NACT (+) *n* = 73	NACT (−) *n* = 19	SMD	NACT (+) Weighted *n* = 73.0	NACT (−) Weighted *n* = 71.3	SMD
Age^a^, year	57.1 ± 12.7	58.5 ± 16.3	0.095	57.1 ± 12.7	55.6 ± 19.7	0.095
Histology^a^						
SCC	60 (82.2)	14 (73.7)	0.206	60.0 (82.2)	57.8 (81.1)	0.027
Other	13 (17.8)	5 (26.3)		13.0 (17.8)	13.4 (18.9)	
cT^a^						
2a1	4 (5.5)	8 (42.1)	1.120	4.0 (5.5)	4.2 (5.8)	0.137
2a2	5 (6.8)	3 (15.8)		5.0 (6.8)	2.7 (3.8)	
2b	64 (87.7)	8 (42.1)		64.0 (87.7)	64.4 (90.4)	
cN^a^						
0	39 (53.4)	11 (57.9)	0.131	39.0 (53.4)	35.6 (50.0)	0.373
1	28 (38.4)	7 (36.8)		28.0 (38.4)	20.9 (29.3)	
2	6 (8.2)	1 (5.3)		6.0 (8.2)	14.8 (20.7)	
PS						
0	71 (97.3)	18 (94.7)	0.409	71.0 (97.3)	70.9 (99.4)	0.262
1	2 (2.7)	0 (0.0)		2.0 (2.7)	0.0 (0.0)	
2	0 (0.0)	1 (5.3)		0.0 (0.0)	0.4 (0.6)	
Tumor size on MRI, mm						
Before NACT	49.6 ± 11.9	40.0 ± 13.1	0.765	49.6 ± 11.9	41.5 ± 11.9	0.682
After NACT	25.0 ± 17.7	NA	NA	25.0 ± 17.7	NA	NA
Response to NACT						
CR	12 (16.4)	NA	NA	12.0 (16.4)	NA	NA
PR	42 (57.5)	NA		42.0 (57.5)	NA	
SD	18 (24.7)	NA		18.0 (24.7)	NA	
PD	0	NA		0	NA	
Duration of surgery, minute	543 ± 141	490 ± 112	0.422	543.5 ± 140.9	478.1 ± 91.1	0.551
Blood loss, mL	1394 ± 701	1227 ± 876	0.211	1394.5 ± 700.7	1267.8 ± 476.9	0.211
Nerve sparing						
None	11 (15.1)	3 (15.8)	0.943	11.0 (15.1)	20.1 (28.1)	0.943
Unilateral	22 (30.1)	0 (0.0)		22.0 (30.1)	0.0 (0.0)	
Bilateral	40 (54.8)	16 (84.2)		40.0 (54.8)	51.2 (71.9)	
(y)pT						
0	3 (4.1)	0 (0.0)	1.122	3.0 (4.1)	0.0 (0.0)	1.264
1a1	5 (6.8)	0 (0.0)		5.0 (6.8)	0.0 (0.0)	
1a2	2 (2.7)	0 (0.0)		2.0 (2.7)	0.0 (0.0)	
1b1	11 (15.1)	1 (5.3)		11.0 (15.1)	7.0 (9.8)	
1b2	6 (8.2)	1 (5.3)		6.0 (8.2)	0.7 (1.0)	
1b3	1 (1.4)	2 (10.5)		1.0 (1.4)	14.8 (20.7)	
2a1	9 (12.3)	3 (15.8)		9.0 (12.3)	2.3 (3.2)	
2a2	4 (5.5)	6 (31.6)		4.0 (5.5)	18.0 (25.3)	
2b	31 (42.5)	6 (31.6)		31.0 (42.5)	28.6 (40.1)	
4	1 (1.4)	0 (0.0)		1.0 (1.4)	0.0 (0.0)	
(y)pN						
0	41 (56.2)	8 (42.1)	0.623	41.0 (56.2)	31.8 (44.6)	0.588
1	25 (34.2)	11 (57.9)		25.0 (34.2)	39.5 (55.4)	
2	7 (9.6)	0 (0.0)		7.0 (9.6)	0.0 (0.0)	
LVSI	31 (42.5)	10 (52.6)	0.428	31.0 (42.5)	38.6 (54.1)	0.277
Positive surgical margins	10 (13.7)	3 (15.8)	0.059	10.0 (13.7)	10.0 (14.0)	0.010
Pathological tumor size, mm	32.8 ± 22.5	49.7 ± 16.1	0.862	32.8 ± 22.5	47.9 ± 13.5	0.811
Adjuvant						
None	2 (2.7)	4 (21.1)	0.677	2.0 (2.7)	21.9 (30.7)	0.872
Chemotherapy	67 (91.8)	15 (78.9)		67.0 (91.8)	49.4 (69.3)	
CCRT	4 (5.5)	0 (0.0)		4.0 (5.5)	0.0 (0.0)	
VD						
None	37 (50.7)	11 (57.9)	0.319	37.0 (50.7)	23.3 (32.7)	0.623
Medication only	21 (28.8)	3 (15.8)		21.0 (28.8)	15.5 (21.8)	
Temporary CIC	12 (16.4)	4 (21.1)		12.0 (16.4)	17.7 (24.8)	
Permanent CIC	3 (4.1)	1 (5.3)		3.0 (4.1)	14.8 (20.7)	
Follow-up period, month	75 [61–90]	95 [78–101]	0.375	75 [61–90]	101 [94–111]	1.113

Categorical valuables were presented as *n* (%). Follow-up periods were presented as median [IQR], and all other continuous valuables were presented as mean ± SD

*IPW* inverse probability weighting, *NACT* neoadjuvant chemotherapy, *SMD* standardized mean difference, *SCC* squamous cell carcinoma, *PS* performance status, *MRI* magnetic resonance imaging, *NA* not applicable, *CR* complete response, *PR* partial response, *SD* stable disease, *PD* progressive disease, *LVSI* lymphovascular space invasion, *CCRT* concurrent chemoradiotherapy, *VD* voiding dysfunction, *CIC* clean intermittent catheterization, *IQR* interquartile range, *SD* standard deviation

^a^Covariates used for IPW

### PFS and OS

**Fig. 3:**
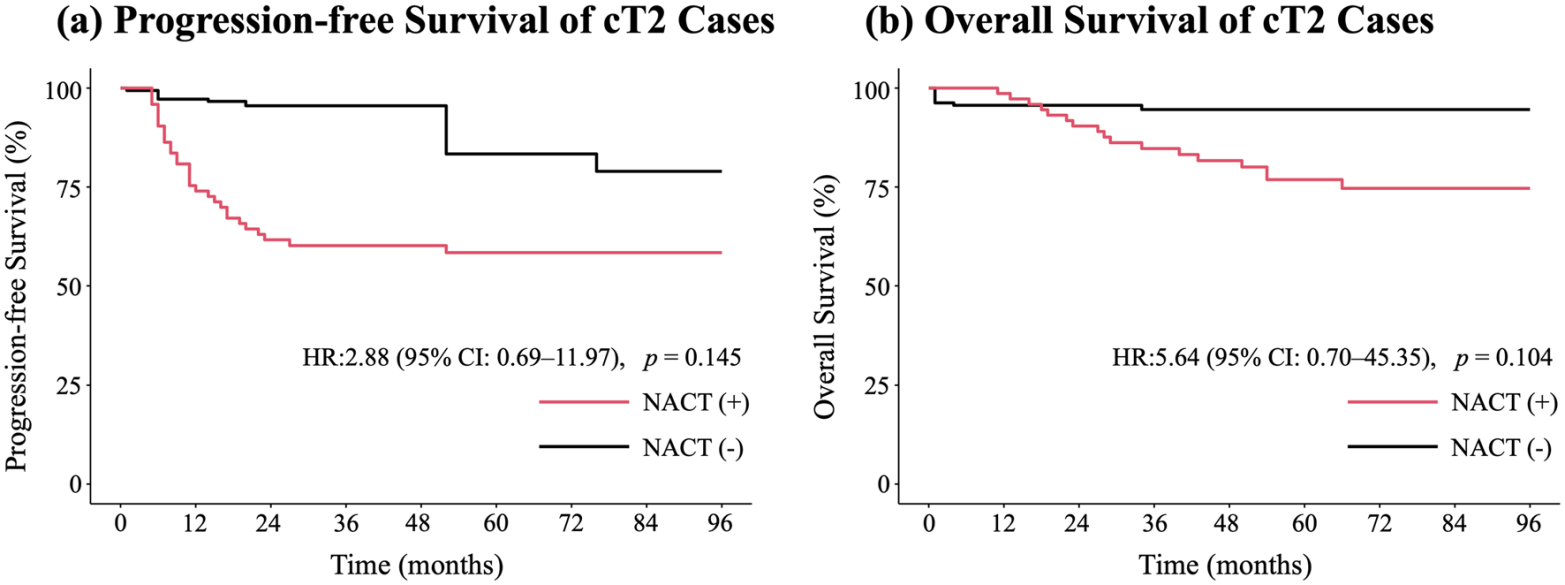
Kaplan–Meier Curves for cT2 Cases After Inverse Probability Weighting. **a** Progression-free Survival of cT2 cases. Although not statistically significant, progression-free survival tended to be worse in the NACT(+) group. The 5-year progression-free survival was 58.4% in the NACT(+) group and 83.3% in the NACT(−) group. **b** Overall Survival of cT2 cases. Although not statistically significant, overall survival tended to be worse in the NACT(+) group. The 5-year overall survival was 76.8% in the NACT(+) group and 94.6% in the NACT(−) group. *HR* hazard ratio, *NACT* neoadjuvant chemotherapy

Weighted Kaplan–Meier curves were generated (Fig. 3). In Cox proportional hazards models additionally adjusted for cT and cN (which remained imbalanced after weighting), with NACT(−) as the reference, PFS showed a non-significant trend toward poorer outcomes in the NACT(+) group (HR 2.88, 95% CI 0.69–11.97; *p* = 0.145; events: NACT(+) 30/73 vs. NACT(−) 6/19). Similar results were observed for OS (HR 5.64, 95% CI 0.70–45.35; *p* = 0.104; events: NACT(+) 18/73 vs. NACT(−) 3/19).

The 5-year PFS was 58.4% (95% CI, 48.0–71.0%) in the NACT(+) group and 83.3% (95% CI, 62.8–100.0%) in the NACT(−) group. The 5-year OS was 76.8% (95% CI, 67.5–87.5%) in the NACT(+) group and 94.6% (95% CI, 86.7–100.0%) in the NACT(−) group.

The sensitivity analysis in which time zero was set to the date of surgery for both groups is shown in Supplementary Fig. S3, and yielded results similar to those of the primary analysis.

### Prognosis according to NACT response

Baseline characteristics stratified by NACT response in the cT2 NACT(+) cohort are shown in Supplementary Table S1. The maximum tumor diameter on MRI before NACT did not differ between groups (mean, 53.9 mm vs. 48.4 mm; *p* = 0.125), whereas the maximum tumor diameter on MRI after NACT was significantly larger in the objective response(−) group than in the objective response(+) group (47.1 mm vs. 17.7 mm; *p* < 0.001). LVSI was also more frequent in the objective response(−) group (83.3% [15/18] vs. 29.6% [16/54]; *p* < 0.001), accompanied by a higher rate of positive surgical margins and a trend toward more advanced ypN stage. However, there was no clear between-group difference in histology.

Kaplan–Meier curves are presented in Supplementary Fig. S4. PFS and OS were significantly worse in the objective response(−) group (*p* < 0.001). The 5-year PFS was 22.2% vs. 69.7% (PFS events, 14/18 vs. 16/54), and the 5-year OS was 31.3% vs. 91.7% (OS events, 12/18 vs. 4/54). In the objective response(−) group, six patients experienced local recurrence and eight experienced distant recurrence.

## Discussion

In this study, we first showed that, among patients with cT1b3 disease, NACT-RH was associated with encouraging long-term outcomes. Although the sample size was limited (*n* = 15), OS remained 100% in the cT1b3 NACT-RH group. When interpreted against a lower-risk reference cohort (cT1b1–1b2, cN0) treated with primary RH, these outcomes provide a descriptive benchmark. Given the structurally different risk profiles between cohorts, this comparison should not be interpreted as evidence of treatment equivalence. Patients with cT1b1–1b2 and cN0 disease had a higher incidence of LVSI than those with cT1b3 disease. LVSI has been reported to be less frequent after NACT than without NACT [16]; however, the observed between-cohort difference in LVSI may reflect a combination of underlying biological differences and the influence of NACT, and the relative contribution of each cannot be determined in our study. Accordingly, this finding should be interpreted with caution.

In contrast, in patients with cT2 disease, we did not observe an added benefit of NACT. Although outcomes appeared poorer in the NACT-treated group, this observation should be interpreted with caution because residual imbalance in cT and cN persisted even after ATT-IPW, representing a potential source of confounding. Notably, PFS and OS were significantly worse among patients with poor NACT response, underscoring the clinical importance of management strategies for NACT-nonresponsive disease.

When our findings in patients with cT1b3 disease are compared with previous reports, NACT-RH in our cohort achieved outcomes that were comparable to, or potentially better than, those reported in the literature. In a cT1b3 subgroup analysis of a Japanese RCT, the 5-year PFS and OS in the NACT-RH group were 60.5% and 78.4%, respectively, and superiority over primary RH was not demonstrated [8]. In another subgroup analysis of an RCT comparing NACT-RH with CCRT, the 5-year DFS after NACT-RH for cT1b3 disease was 74.0%, with outcomes reported to be comparable to those of CCRT [17]. Taken together, these data do not necessarily exclude NACT-RH as a treatment option when restricted to patients with cT1b3 disease. Although the sample size in our study was limited and this analysis was hypothesis-generating in nature, we observed PFS and OS that were at least comparable to those previously reported, suggesting that NACT-RH may be effective for cT1b3 disease.

In addition, only one patient (6.7%) experienced local recurrence among the 15 cT1b3 patients treated with NACT-RH, and the recurrence was successfully controlled surgically. Choosing NACT-RH as the initial treatment may also preserve subsequent therapeutic options: surgical management at recurrence may be more feasible than in-field recurrence after definitive RT, and salvage CCRT remains available [18, 19]. Moreover, grade ≥ 3 late toxicities have been reported in 13% of patients treated with primary CCRT, including urinary and gastrointestinal events [20]. Considering this toxicity profile, NACT-RH may remain a reasonable initial treatment to consider for selected patients with cT1b3 disease.

In contrast, for patients with cT2 disease, the utility of NACT was not supported; in our study, outcomes tended to be poorer among patients who received NACT. Although baseline characteristics were adjusted as much as possible, no incremental benefit of NACT was observed. In a cT2a2–2b subgroup analysis of a Japanese RCT, the 5-year PFS and OS were reported as 59.3% and 65.3% in the NACT-RH group, respectively, and 58.4% and 69.5% in the primary RH group [8]. In our study, outcomes in the NACT-RH group were broadly consistent with these data; however, outcomes in the primary RH group were particularly favorable, which may have made NACT appear to have a negative impact on prognosis. Among patients who underwent primary RH, more than 20% were classified as cN2 in the weighted cohort; however, no pathological lymph node metastases corresponding to pN2 were identified. This absence of pN2 disease may have contributed to the favorable prognosis observed in the NACT(−) group. Moreover, in an RCT comparing NACT-RH with CCRT, DFS was comparable between the two strategies in patients with cT1b3–2a disease, whereas DFS was significantly worse with NACT-RH in patients with cT2b disease [17]. Taken together, these findings suggest that the indication for NACT-RH should be carefully considered, particularly in patients with cT2b disease. Importantly, this comparison is statistically underpowered, particularly because the primary RH group was small, and the resulting CIs were very wide, indicating substantial imprecision. Therefore, our estimates do not support definitive conclusions regarding comparative effectiveness of NACT-RH in cT2 disease, and the observed trends should be interpreted as hypothesis-generating.

One potential explanation for the observed unfavorable outcomes associated with NACT in patients with cT2 disease is that prognosis is particularly poor among those with a poor response to NACT. Because NACT inevitably delays definitive surgery, nonresponding tumors may progress during this interval. Parametrial involvement is considered a risk factor for distant recurrence [21], and in patients with cT2b disease, lack of chemosensitivity may be associated with an increased risk of distant failure. In addition, large tumor size has been linked to local recurrence [21], suggesting that nonresponding tumors may also be disadvantaged in terms of local control. In our study, all 18 patients who did not achieve an objective response to NACT in the cT2 cohort had cT2b disease. Therefore, because all cases without an objective response were cT2b, the poor prognosis in this group likely reflects a combination of cT2b-associated adverse biology and limited chemosensitivity. Accordingly, the absence of an objective response may be useful as a practical indicator of a higher-risk subset within cT2 disease, without implying that outcomes are determined by chemosensitivity alone.

These findings suggest stage-dependent suitability of NACT-RH among patients eligible for RH, and highlight the need to consider NACT-RH alongside other multimodal strategies, including CCRT. In recent years, regimens combining CCRT with immune checkpoint inhibitors (ICI) have been introduced, making it an important clinical question how NACT-RH should be positioned relative to these approaches. In KEYNOTE-A18, the addition of pembrolizumab to standard CCRT, followed by maintenance therapy, demonstrated an incremental PFS benefit of ICI [22], which may further increase the use of CCRT in clinical practice. However, in subgroup analyses, the HR for PFS was 0.58 (95% CI, 0.42–0.80) for cT3 or higher disease, whereas no clear benefit was observed for cT2 or lower disease (HR 0.91, 95% CI, 0.63–1.31). Although subgroup findings are not definitive, these results may indicate that the benefit of intensifying CCRT is not necessarily greater in stages where RH is a feasible option.

On the other hand, ICI-based combinations are also being explored in the neoadjuvant setting. The NACI study, a phase II trial, is currently evaluating NACT combined with an ICI, camrelizumab, for locally advanced cervical cancer. An interim analysis reported an objective response of 98%, comprising complete response in 19% and partial response in 79% [23]. By contrast, objective response rates for conventional NACT without ICI vary by regimen but have generally been reported to be approximately 60–80% [8, 24–26], making the NACI findings noteworthy. Further validation is warranted; however, NACT regimens incorporating ICI may become more widely adopted in the future. Notably, the trial protocol assigned RH for responders and CCRT for nonresponders. This response-adapted strategy—selecting definitive local therapy according to NACT response—aligns with our observation that poor response to NACT is associated with unfavorable NACT-RH outcomes, and may represent a promising approach to improve clinical outcomes.

This study has several limitations. First, as a single-center retrospective cohort study, treatment decision-making and the intensity of follow-up were individualized, and residual unmeasured confounding and information bias cannot be fully excluded. Second, although IPW was used to adjust for measured confounders, imbalance in cT and cN persisted after weighting in the cT2 analysis, making it difficult to rule out residual confounding. Third, the sample size and number of events in each analysis were limited, raising concerns regarding statistical power and the stability of the estimates. This limitation was most pronounced in the cT2 analysis, where the small control group and residual imbalance in cT and cN after ATT-IPW resulted in very wide confidence intervals, reflecting limited statistical precision and insufficient power to draw definitive comparative conclusions. Fourth, because adjuvant chemotherapy was generally planned as part of the treatment strategy when NACT was administered, our results do not isolate the effect of the treatment choice between NACT-RH and primary RH alone. Fifth, the limited sample size precluded stratified analyses by histologic subtype. Sixth, as the study population was limited to patients who ultimately underwent RH, our estimates may be subject to an immortal time bias in the NACT-RH group, potentially favoring NACT-RH. We addressed this issue by conducting a sensitivity analysis with time zero set to the date of surgery in both groups (Supplementary Figs. S1 and S3). Finally, because this study reflects outcomes from a Japanese high-volume center where the Okabayashi RH is standard, caution is warranted when generalizing these findings to other institutions or healthcare settings.

A key strength of this study is that it evaluated outcomes of NACT-RH at a single high-volume center with a relatively uniform surgical strategy and perioperative management, thereby minimizing variability in surgical practice. In addition, we demonstrated prognostic differences according to NACT response, which may inform future treatment selection—particularly if NACT regimens incorporating ICI achieve very high response rates and enable response-adapted decision-making.

In conclusion, NACT-RH may represent a potentially effective treatment option for patients with cT1b3 disease; however, its benefit in cT2 disease, particularly cT2b, remains uncertain, and careful patient selection is warranted. If consistently high response rates can be achieved with NACT incorporating ICI, NACT-RH may be more widely accepted as a standard treatment in the future.

## Supplementary Information

Below is the link to the electronic supplementary material.

Supplementary file1 (PDF 731 KB)

Supplementary file2 (PDF 63 KB)

Supplementary file3 (PDF 771 KB)

Supplementary file4 (PDF 572 KB)

Supplementary file5 (DOCX 18 KB)

## Data Availability

The data supporting the findings of this study are available from the corresponding author upon reasonable request.
