# Supplementary figures and images for: Evaluation of neoadjuvant chemotherapy followed by radical hysterectomy in cervical cancer: a single-center study
Source: Int J Clin Oncol. 2026 Mar 4;31(4):762–72. doi: 10.1007/s10147-026-02998-0 (PMC13018027; doi:10.1007/s10147-026-02998-0)

**(a) Progression-free Survival of cT1 Cases**

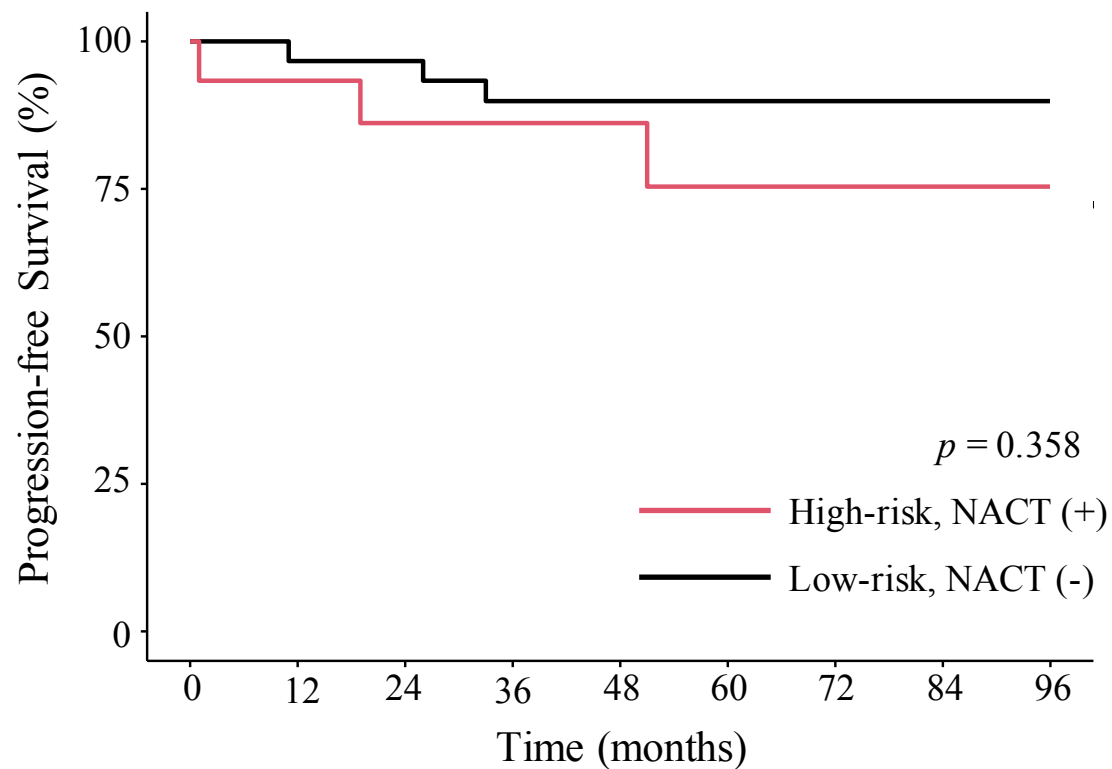

**(b) Overall Survival of cT1 Cases**

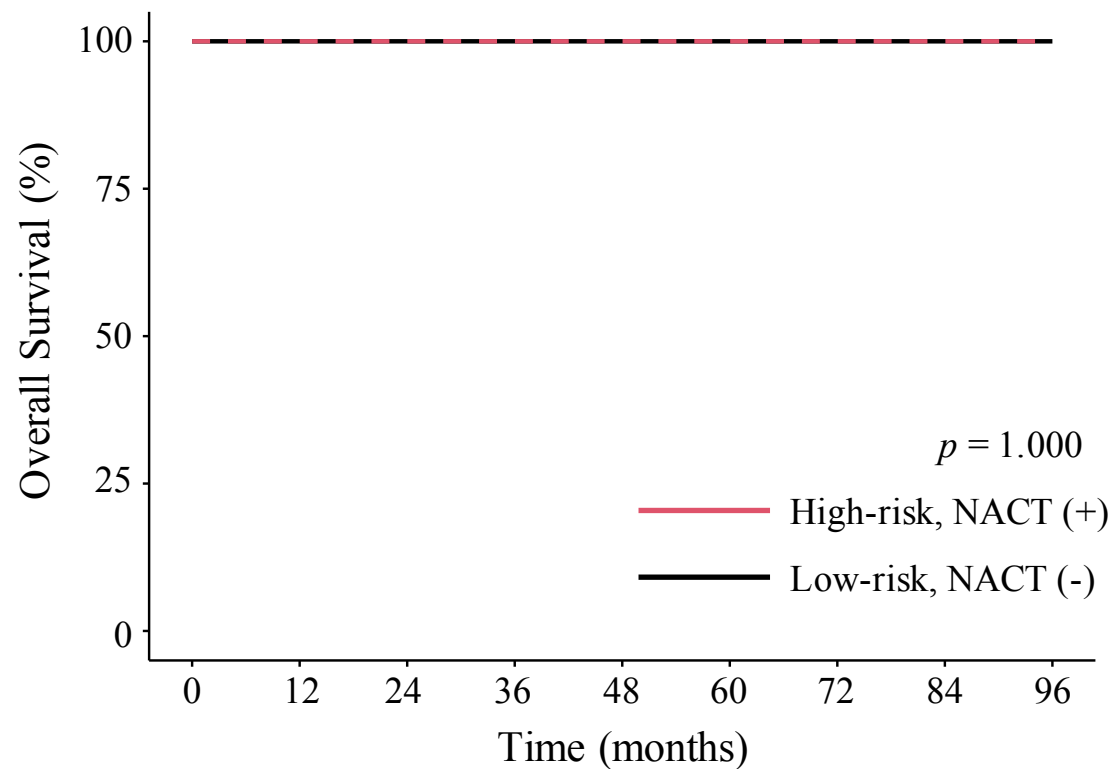

Supplement: Supplementary file 1 — Supplementary file1 (PDF 731 KB) [file 10147_2026_2998_MOESM1_ESM.pdf]

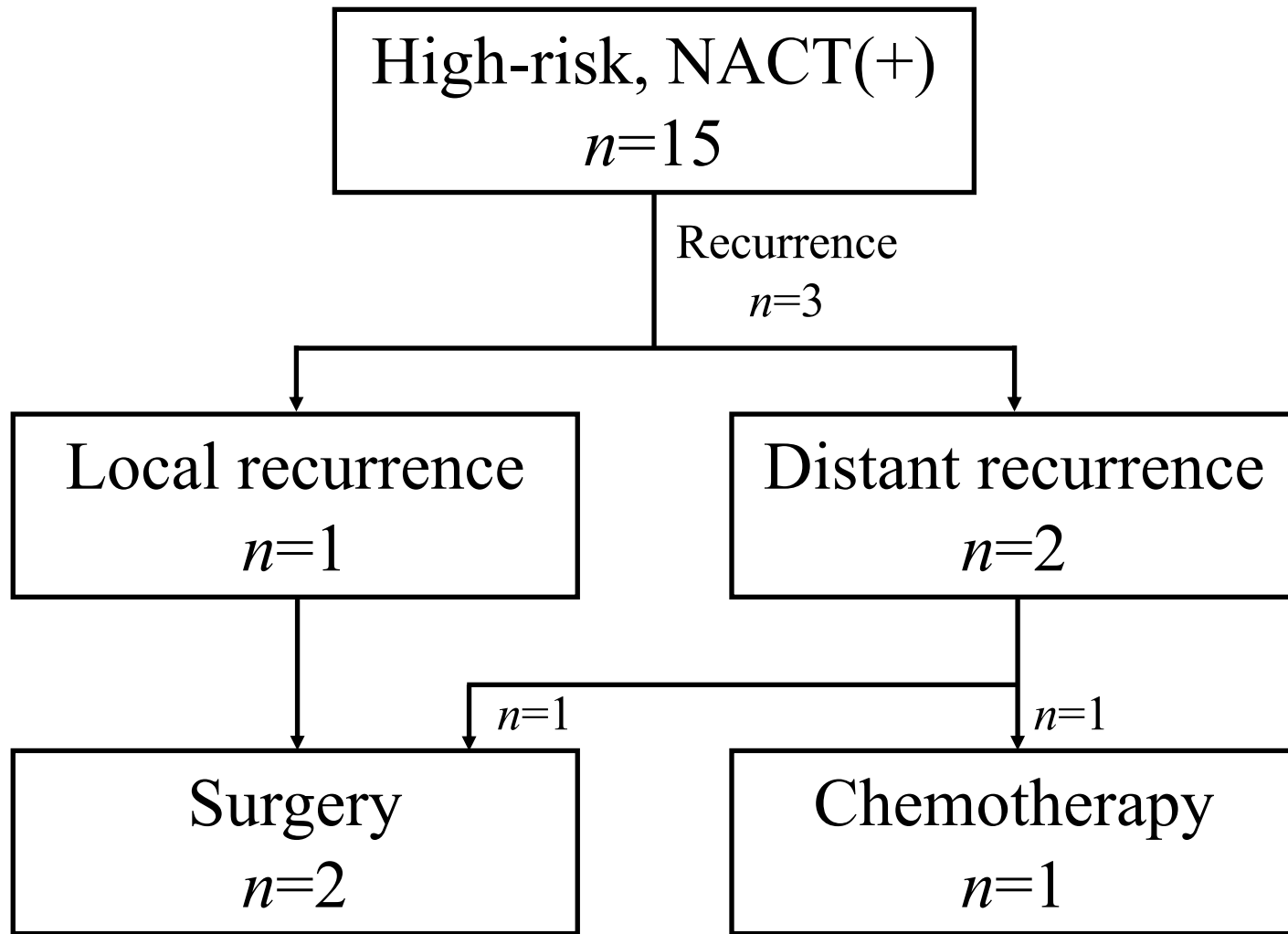

No deaths were observed.

Supplement: Supplementary file 2 — Supplementary file2 (PDF 63 KB) [file 10147_2026_2998_MOESM2_ESM.pdf]

**(a) Progression-free Survival of cT2 Cases**

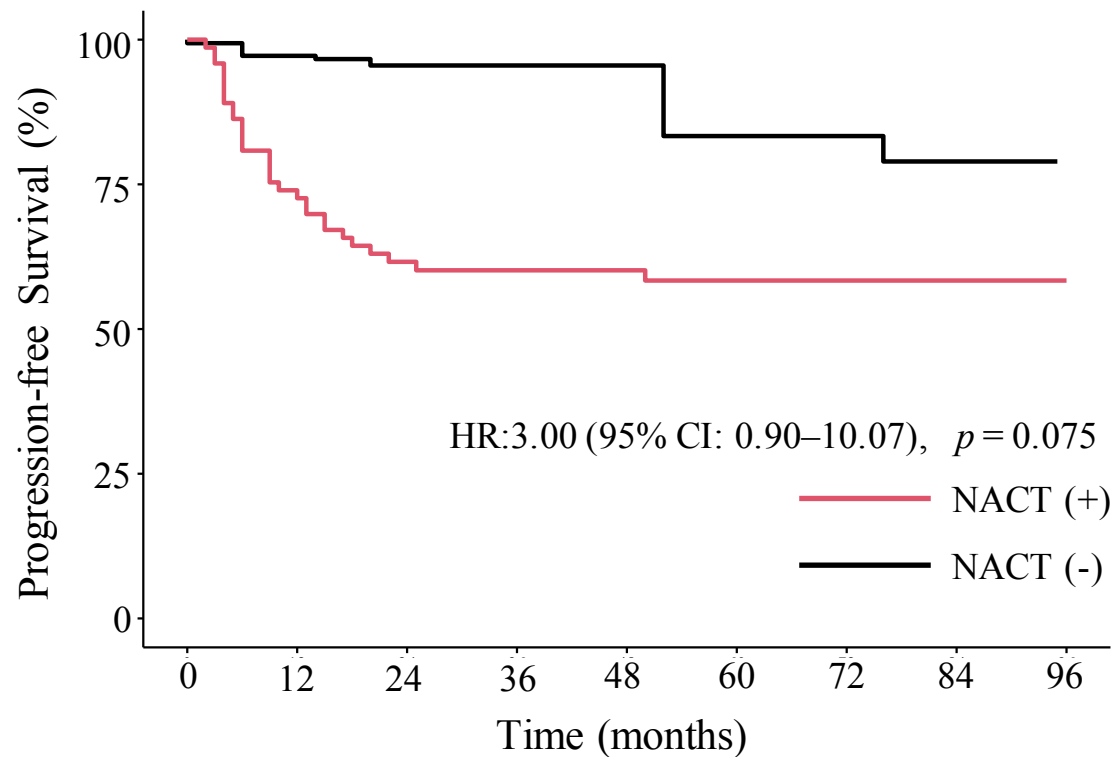

**(b) Overall Survival of cT2 Cases**

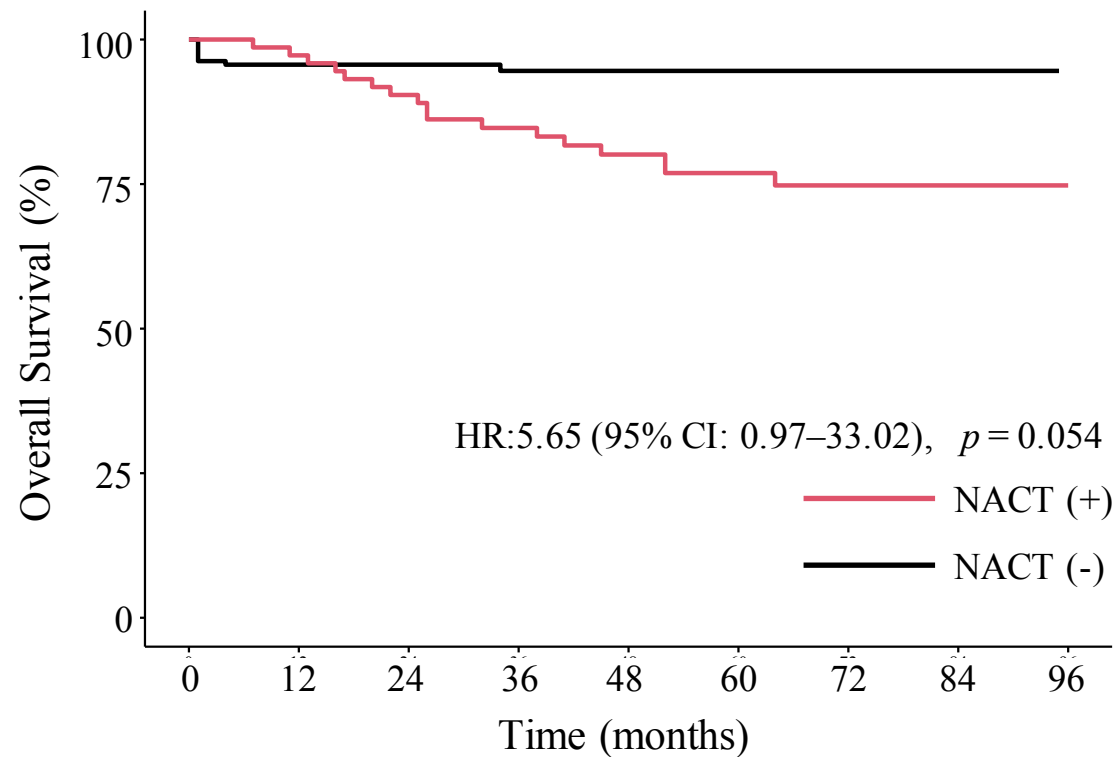

Supplement: Supplementary file 3 — Supplementary file3 (PDF 771 KB) [file 10147_2026_2998_MOESM3_ESM.pdf]
