# Supplementary material for: Evaluation of neoadjuvant chemotherapy followed by radical hysterectomy in cervical cancer: a single-center study
Source: Int J Clin Oncol. 2026 Mar 4;31(4):762–72. doi: 10.1007/s10147-026-02998-0 (PMC13018027; doi:10.1007/s10147-026-02998-0)
Supplement: Supplementary file 4 — Supplementary file4 (PDF 572 KB) [file 10147_2026_2998_MOESM4_ESM.pdf]

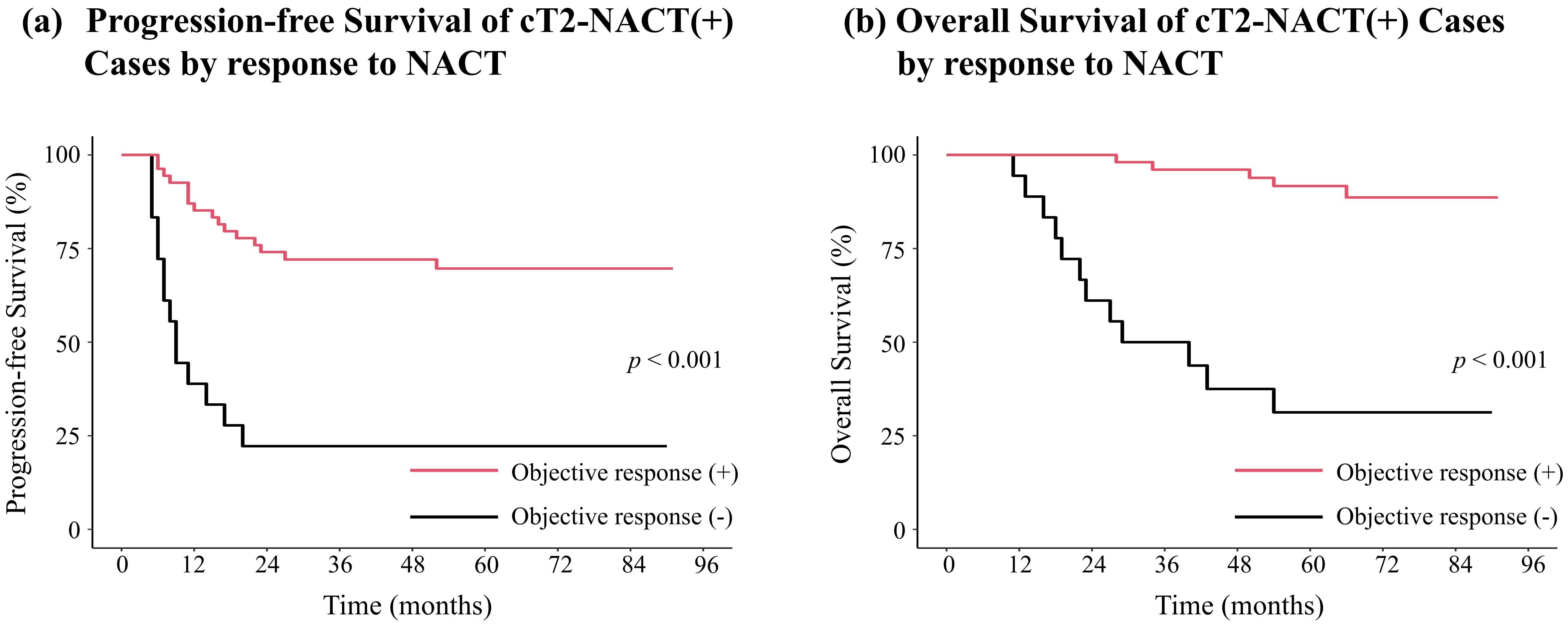

(a) Progression-free Survival of cT2-NACT(+) Cases by response to NACT
Progression-free Survival (%)
100
75
50
25
0
p < 0.001
Objective response (+)
Objective response (-)
0
12
24
36
48
60
72
84
96
Time (months)
(b) Overall Survival of cT2-NACT(+) Cases by response to NACT
Overall Survival (%)
100
75
50
25
0
p < 0.001
Objective response (+)
Objective response (-)
0
12
24
36
48
60
72
84
96
Time (months)
