# Supplementary material for: Evaluation of neoadjuvant chemotherapy followed by radical hysterectomy in cervical cancer: a single-center study
Source: Int J Clin Oncol. 2026 Mar 4;31(4):762–72. doi: 10.1007/s10147-026-02998-0 (PMC13018027; doi:10.1007/s10147-026-02998-0)
Supplement: Supplementary file 5 — Supplementary file5 (DOCX 18 KB) [file 10147_2026_2998_MOESM5_ESM.docx]

Supplementary Table S1. Baseline Characteristics and Surgical Details of cT2-NACT (+) Patients According to Response to NACT

|  | Objective response (+)  *n*=54 | Objective response (-)  *n*=18 | *p* |
| --- | --- | --- | --- |
| Age, year | 58.6±13.1 | 53.0±11.3 | 0.089 |
| Histology: SCC  Other | 45 (83.3)  9 (16.7) | 14 (77.8)  4 (22.2) | 0.774 |
| cT: 2a1  2a2  2b | 4 (7.4)  5 (9.3)  45 (83.3) | 0 (0.0)  0 (0.0)  18 (100.0) | 0.3936 |
| cN: 0  1  2 | 32 (59.3)  18 (33.3)  4 (7.4) | 6 (33.3)  10 (55.6)  2 (11.1) | 0.208 |
| PS: 0  　 1 | 53 (98.1)  1 (1.9) | 17 (94.4)  1 (5.6) | 0.456 |
| Tumor size on MRI, mm  before NACT  after NACT | 48.4±11.2  17.7±11.7 | 53.9±13.2  47.1±14.1 | 0.125  <0.001 |
| Response to NACT: CR  PR  SD  PD | 12 (22.2)  42 (77.8)  0 (0.0)  0 (0.0) | 0 (0.0)  0 (0.0)  18 (100.0)  0 (0.0) | <0.001 |
| Duration of surgery, minute | 519±114 | 630±172 | 0.018 |
| Blood loss, mL | 1284±630 | 1695±830 | 0.066 |
| Nerve sparing: None  unilateral  bilateral | 3 (5.6)  19 (35.2)  32 (59.3) | 7 (38.9)  3 (16.7)  8 (44.4) | 0.002 |
| ypT: 0  1a1  1a2  1b1  1b2  1b3  2a1  2a2  2b  4 | 3 (5.6)  5 (9.3)  2 (3.7)  11 (20.4)  6 (11.1)  0 (0.0)  7 (13.0)  3 (5.6)  17 (31.5)  0 (0.0) | 0 (0.0)  0 (0.0)  0 (0.0)  0 (0.0)  0 (0.0)  1 (5.6)  1 (5.6)  1 (5.6)  14 (77.8)  1 (5.6) | 0.005 |
| ypN: 0  1  2 | 34 (63.0)  16 (29.6)  4 (7.4) | 6 (33.3)  9 (50.0)  3 (16.7) | 0.121 |
| LVSI | 16 (29.6) | 15 (83.3) | <0.001 |
| Positive surgical margins | 4 (7.4) | 6 (33.3) | 0.025 |
| Pathological tumor size, mm | 24.9±17.0 | 57.1±20.6 | <0.001 |
| Adjuvant: None  Chemotherapy  CCRT | 2 (3.7)  52 (96.3)  0 (0.0) | 0 (0.0)  14 (77.8)  4 (22.2) | 0.009 |
| VD: None  Medication only  Temporary CIC  Permanent CIC | 27 (50.0)  17 (31.5)  7 (13.0)  3 (5.6) | 9 (50.0)  4 (22.2)  5 (27.8)  0 (0.0) | 0.633 |
| Follow-up period, month | 73 [61–90] | 81 [59–90] | 0.584 |

Categorical valuables were presented as *n* (%). Follow-up periods were presented as median [IQR], and all other continuous valuables were presented as mean ± SD.

NACT, neoadjuvant chemotherapy; SCC, squamous cell carcinoma; PS, performance status; MRI, magnetic resonance imaging; CR, complete response; PR, partial response; SD, stable disease; PD, progressive disease; LVSI, lymphovascular space invasion; CCRT, concurrent chemoradiotherapy; VD, voiding dysfunction; CIC, clean intermittent catheterization; IQR, interquartile range; SD, standard deviation
